# In-Cell Intrabody Selection from a Diverse Human Library Identifies C12orf4 Protein as a New Player in Rodent Mast Cell Degranulation

**DOI:** 10.1371/journal.pone.0104998

**Published:** 2014-08-14

**Authors:** Elsa Mazuc, Laurence Guglielmi, Nicole Bec, Vincent Parez, Chang S. Hahn, Caroline Mollevi, Hugues Parrinello, Jean-Pierre Desvignes, Christian Larroque, Ray Jupp, Piona Dariavach, Pierre Martineau

**Affiliations:** 1 IRCM, Institut de Recherche en Cancérologie de Montpellier, Montpellier, France; 2 INSERM, U896, Montpellier, France; 3 Université Montpellier1, Montpellier, France; 4 ICM, Institut régional du Cancer Montpellier, Montpellier, France; 5 Sanofi-Aventis, Bridgewater, New Jersey, United States of America; 6 MGX-Montpellier GenomiX, Institut de Génomique Fonctionnelle, Montpellier, France; 7 Université Montpellier2, Montpellier, France; Technical University of Braunschweig, Germany

## Abstract

The high specificity of antibodies for their antigen allows a fine discrimination of target conformations and post-translational modifications, making antibodies the first choice tool to interrogate the proteome. We describe here an approach based on a large-scale intracellular expression and selection of antibody fragments in eukaryotic cells, so-called intrabodies, and the subsequent identification of their natural target within living cell. Starting from a phenotypic trait, this integrated system allows the identification of new therapeutic targets together with their companion inhibitory intrabody. We applied this system in a model of allergy and inflammation. We first cloned a large and highly diverse intrabody library both in a plasmid and a retroviral eukaryotic expression vector. After transfection in the RBL-2H3 rat basophilic leukemia cell line, we performed seven rounds of selection to isolate cells displaying a defect in FcεRI-induced degranulation. We used high throughput sequencing to identify intrabody sequences enriched during the course of selection. Only one intrabody was common to both plasmid and retroviral selections, and was used to capture and identify its target from cell extracts. Mass spectrometry analysis identified protein RGD1311164 (C12orf4), with no previously described function. Our data demonstrate that RGD1311164 is a cytoplasmic protein implicated in the early signaling events following FcεRI-induced cell activation. This work illustrates the strength of the intrabody-based in-cell selection, which allowed the identification of a new player in mast cell activation together with its specific inhibitor intrabody.

## Introduction

Mast cells and basophils are key effector cells in IgE-associated immediate hypersensitivity and allergic disorders. Upon FcεRI crosslinking initiated by the binding of antigen-IgE complexes, cell activation results in downstream events that lead to the secretion of three classes of mediators: (a) the extracellular release of preformed mediators stored in cell cytoplasmic granules, by a process called degranulation; (b) the de novo synthesis of proinflammatory lipid mediators; and (c) the synthesis and secretion of many growth factors, cytokines, and chemokines. This IgE-dependent release of mediators begins within minutes of antigen challenge and leads to certain acute allergic reactions such as anaphylaxis and acute attacks of atopic asthma [1].

The majority of drugs currently used to treat allergic disorders target only a single mediator released by mast cells. Examples include antihistamine H1 receptor antagonists, leukotriene modifiers, and steroids that predominantly inhibit mast-cell mediator production. More recently, protein therapies have permitted alternative approaches in addition to drug therapies. In this respect, an important treatment for allergic conditions is the recombinant humanized IgG monoclonal antibody Omalizumab, which binds selectively to human IgE and inhibit the production and release of all mast cell mediators by antagonizing IgE action. Although this biologic is highly effective, it is difficult and expensive to manufacture and administer.

An alternative that has gained significant attention in recent years is to target key enzymes involved in the signal transduction pathways initiated following FcεRI crosslinking. Mast cell activation results from the transient perturbation of an active balance between positive and negative signals that is consequent to engagement of membrane receptors. Classically, kinases and phosphatases have been viewed as the effectors of positive and negative signals, respectively. FcεRI mainly trigger positive signals by recruiting tyrosine kinases and signalosomes into which signaling molecules assemble [2].

In the past decade, one of the compelling targets for the treatment of allergic and autoimmune disorders was the Spleen tyrosine kinase (Syk), a key mediator of immunoreceptor signaling [3]. Many pharmaceutical companies as well as academic institutions have been involved in the development of small-molecule inhibitors of Syk that target the conserved ATP binding site within the catalytic domain of the kinase. But due to the similarities of the ATP pocket structures among different kinases, the ATP-binding site inhibitors of Syk affect multiple tyrosine kinases and have off-target effects that lead to undesirable side effects [4]. For these reasons, clinical trials using systemic modes of administration of Syk inhibitors were abandoned in favor of local modes of administration. Examples are the compound R112, the first Syk inhibitor to enter clinical studies developed by Rigel as an intranasal administration for seasonal allergic rhinitis [5] and R343, an inhaled formulation for the treatment of allergic asthma (Pfizer) [6].

In our previous studies, we devised an approach to identify protein-protein interaction and allosteric inhibitors of Syk instead of targeting its catalytic site. Our goal was to improve the selectivity and the safety profiles of Syk inhibitor drug candidates by selecting drugs targeting the SH2 domains of Syk. To achieve this, we developed an antibody displacement assay to convert an intrabody directed against the SH2 domain of Syk into chemical drugs [7], [8]. The isolated molecules recapitulated the intrabody effects in cell cultures and were able to block the anaphylactic shock when administrated orally in animal models [7]. This led to the identification of several scaffolds as potential starting points for the development of new classes of non-enzymatic inhibitors of Syk with minimal off-target effects [7], [8].

The anti-Syk inhibitory intrabody used in the above studies was selected from a two-step process: a) *in vitro* screen of a phage display library against a recombinant Syk protein; and b) intracellular expression of the isolated antibody fragments in mammalian cells to test their inhibitory potential [9]. However, all targets may not be identified beforehand and it is tempting to envision a direct selection of intrabodies in cells. The use of an intrabody library as a target discovery platform has been suggested almost 20 years ago by Dr. A. Cattaneo and co-workers [10], [11]. In a pioneering experiment, they demonstrated the rescue of an antiviral neutralizing intrabody diluted within a polyclonal repertoire. Although this was obtained in a model system with a limited diversity, this paved the way to a direct selection of a diverse repertoire of antibody fragments based on a selectable cell phenotype [12].

We report here the first application of such a strategy for the identification of new therapeutic targets in the field of allergy and inflammation. The Intrabody-based Phenotypic Screen (IBPheS) is based on the intracellular expression of a highly diverse antibody fragment library in eukaryotic cells and the selection of antibody fragments associated with the desired phenotype. Because this method is based on the intrabody-target interaction, it results in the co-selection of a target with its companion intrabody. This ensures that the fished targets are accessible to in-cell modulation (inhibition or activation) and thus may represent a “druggable” class of proteins. In the complex biological system of FcεRI-induced cell activation, the IBPheS approach led to the discovery of a protein of yet unknown function implicated in mast cell degranulation.

## Materials and Methods

### Reagents and Antibodies

Antibodies were obtained from Santa Cruz Biotechnology (Santa Cruz, CA, USA), with the exception of anti-p44/42 MAP Kinase antibody and the following phospho-specific antibodies which were obtained from Cell Signaling Technologies (Danvers, MA, USA): Phospho Syk (Tyr525/526); Phospho-p44/42 MAPK (Erk1/2) (Thr202/Tyr204); Phospho-SAPK/JNK (Thr183/Tyr185); Phospho-p38 MAP Kinase (Thr180/Tyr182); Phospho-Src Family (Tyr416); Phospho-NFkB (Ser536); Phospho-Gab2 (Tyr452); Phospho-PLCγ2 (Tyr1217); Phospho-Akt (Ser473). The anti-human C12orf4 antibody and all reagents were obtained from Sigma-Aldrich (St Louis, MO, USA). Antiphosphotyrosine mAb 4G10 was purchased from Upstate Biotechnology (Millipore, MA, USA). Alexa 488 conjugated anti-mouse IgG and Alexa 594 conjugated anti-rabbit IgG antibodies were purchased from Jackson ImmunoResearch laboratories (West Grove, PA, USA).

### Murine bone marrow derived mast cells

To generate BMMCs, femur bones from C57BL/6 female mice (4–6 weeks old, Charles River) were isolated and progenitor cells were flushed out using a sterile protocol and cultured in Opti-MEM medium supplemented with 10% foetal calf serum (FCS), 4 mM glutamine, 100 units/ml of penicillin, 100 µg/ml of streptomycin, and 50 mM 2-mercaptoethanol together with 1 ng/ml of recombinant murine IL-3 (Biolegend). All experiments were performed in compliance with the French guidelines for experimental animal studies, and protocols were approved by the Institute of Cancer Research Ethics Committee (agreement no. B34-172-27). All reasonable efforts were made to ameliorate suffering, including anesthesia for painful procedures.

### Cell culture

Culture media were obtained from Gibco (Life Technologies Ltd, Paisley, UK). Rat basophilic leukemia cell line RBL-2H3 was obtained from the ATCC (Manassas, VA, USA) and cultured in DMEM medium supplemented with 15% FCS and antibiotics. Line 293T (or HEK-T) cells were maintained in culture in DMEM medium supplemented with 10% FCS and antibiotics. The murine hybridoma 2682-1 producing anti-2,4-dinitrophenyl (DNP) IgE mAb was maintained in culture in DMEM medium supplemented with 10% FCS and antibiotics, and its culture supernatants, containing 1 µg/ml of IgE (measured by ELISA), were filtered and preserved at −20°C.

### Intrabody library construction

Expression vector pEF/myc/cyto (Invitrogen, Life Technologies Ltd, Paisley, UK) was used to express the scFv library in the cytoplasm of RBL-2H3 cells. In order to clone the scFv library into this vector, VHpool and VLpool sub-libraries, which are the source of the diversity of the CDR3 loops in a previously described library [13], were assembled by PCR, cloned into the NcoI-NotI linearized vector, and transformed in E. coli. Library diversity was estimated to 10^9^ by counting the obtained number of clones. An aliquot corresponding to 40 times the diversity of the library was used to prepare the recombinant plasmid DNA using the Nucleobond Xtra Maxi kit (Macherey Nagel) that was subsequently used for the transfection of the RBL-2H3 cell line.

For the expression of the scFv library by retroviral infection, the scFv library was inserted between SfiI and NotI sites in pMSCVhygSN-EGFP vector as described [14]. The estimated diversity of the library was 2×10^8^. Recloning steps after rounds 3 and 5 during selection were performed by amplification of the inserted scFv from the chromosome using pMSCV.for (cgttcgaccccgcctcg) and EGFP-N.back (cgtcgccgtccagctcgaccag) primers using Phusion polymerase, followed by SfiI-NotI recloning in the same retroviral vector.

### Plasmid transfection of the scFv library

RBL-2H3 cells (5×10^6^) were mixed with 50 µg of plasmid and transferred in a 4 mm electroporation cuvette (BioRad, Hercules, CA, USA). The electroporation was performed with a Gene Pulser I (BioRad) at 310 V and 960 µF capacitance. 3×10^8^ cells were electroporated for the first selection round, and 1×10^8^ cells for each subsequent round (cell survival rate of 20%). For stable clone generation, cells were grown in culture medium supplemented with 1 mg/ml of geneticin (G418, Gibco).

### Retroviral infection of the scFv library

Retroviral particles were produced in 293T cells using manufacturer instructions using an amphotropic envelop gene (VSV-G). Culture supernatants containing retroviral particles were collected, filtered, and used for the infection of 4×10^7^ RBL-2H3 for the first selection round, and 7×10^6^ cells for the recloning step after rounds 3 and 5. 48h post-infection, culture medium was replaced with fresh medium supplemented with 1mg/ml of hygromycin B (Invitrogen) as selecting agent.

### Cell activation, Annexin V staining and cell sorting

Cells were incubated overnight at 37°C with anti-DNP IgE hybridoma supernatant at a final IgE concentration of 0.5 µg/ml. Cells were washed once with RPMI, then with Tyrode buffer (10 mM HEPES pH 7.4, 130 mM NaCl, 5 mM KCl, 1 mM CaCl2, 1 mM MgCl2, 5.6 mM glucose, and 0.01% BSA). Cells were activated in Tyrode buffer with 100 ng/ml of DNP-KLH (keyhole limpet hemocyanin conjugated DNP, Sigma-Aldrich) at 37°C in the dark, for 45 minutes. Cells were subsequently washed in ice-cold Tyrode buffer.

For the Annexin V-APC (Becton Dickinson Biosciences, San Jose, CA, USA) staining, 100 µl Annexin V-APC were added to 2×10^6^ cells (in 500 µl), placed 30 min on ice in the dark. The cells were then labeled with 20 µg/ml Propidium Iodide 3 minutes prior to FACS analysis. Analysis and sorting by flow cytometry were performed using FACS Aria cell sorter (Becton Dickinson, Franklin Lakes, NJ, USA). For plasmid library selection, 2×10^8^ and 10^7^ cells were sorted at the first round and following rounds respectively. For retroviral selection, 4×10^7^ and 10^7^ cells were sorted at the first round and following rounds respectively.

### High throughput sequencing

The genomic DNA of 10^6^ cells was extracted and the scFv gene amplified using primers HTSVHFR3.for (nnctgtttattactgtgtgaga) and HTSVLFR4.back (nncttggtccctccgccgaa) that hybridize to the 3' end of the FR3 of the VH and the 5' end of the FR4 of the VL respectively, and contained a 2-base index (nn in the sequence) for sequence multiplexing. This resulted in a band of 450 bp bordered by the VH and VL CDR3 regions with 18–20 bases from the flanking FR regions and a 2-base index.

Library construction was performed using the ChIPseq sample preparation kit from Illumina (IP-102–1001, San Diego, CA, USA). Briefly, 120 ng of the PCR product were repaired using T4 DNA polymerase, Klenow DNA polymerase and T4 PolyNucleotide Kinase. An A was added at each 3' end followed by ligation of Illumina's adapters. A size selection was performed on a 2% agarose gel in the 500 to 900pb range followed by an 18 cycles PCR amplification. Once purified, the library was validated using a DNA1000 chip on a BioAnalyzer (Agilent Technologies, Santa Clara, CA, USA). Library was denatured using NaOH, hybridized on the flow cell at a concentration of 4 pM and clusterized. A 100-cycle single read sequencing was performed according to the manufacturer's instructions.

Image analyses and base calling were performed using the HiSeq Control Software (HCS 1.3.8) and RTA component (RTA 1.10.36). Using a perl script, libraries were sorted using the first 2 bases indexes (no mismatch allowed) and the next 18 to 20 bases (corresponding to the FR regions of the 2 primers) allowing one mismatch. Count of various random parts is performed using a perl script on the first 25 bases.

### Sequence analysis

All analyses were done using the R statistical environment (http://www.R-project.org). Only the 9493 VH DNA sequences corresponding to a full CDR3 loop and present in both the naive and the final round 7 libraries were used. A two-class unpaired SAM was implemented, using the R package “samr” (http://CRAN.R-project.org/package=samr) [15]. In order to identify VH sequences enriched during the selection, we compared naive and round 3 (before and after the recloning step) to rounds 5, 7 and 8 libraries. If False Discovery Rate was <0.05, VH were considered as significantly enriched. The identified 2568 sequences were filtered by keeping those that did not contain a stop codon and whose frequency regularly increased during the selection (Naive < Round 3< Round 5< Round 7), resulting in 529 VH. Finally we selected 108 VH that were enriched at least 100-fold during the selection process (max(Round 7,Round 8) >100× naive).

Sequences were translated and aligned using IMGT numbering [16]. Distances between sequences were calculated by giving a value of 1 if loop lengths were different and a value of (% of dissimilarity) when the two loops were of the same length (Normalized Hamming distance). Hierarchical unsupervised clustering was performed using the hclust method of R using the “complete” agglomeration method.

### Measurement of β-hexosaminidase release

RBL-2H3 cells were seeded at 10^5^ cells per well in 96-well culture plates. After 24 hours, adherent cells were incubated overnight with anti-DNP IgE (0.5 µg/ml), and activated in Tyrode buffer containing 50 ng/ml of DNP-KLH for 45 minutes at 37°C, as described [17]. The release of β-hexosaminidase in the supernatant (S1) and the unreleased fraction (S2) were measured using a chromogenic substrate (p-dinitrophenyl-N-acetyl-β-D-glucosaminidase, SIGMA). The percentage of β-hexosaminidase release was calculated according to the ratio: S1/(S1+S2) ×100, and expressed as a percentage of the β-hexosaminidase release of an RBL-2H3 cell line expressing an irrelevant antibody.

### Measurement of TNFα secretion

RBL-2H3 cells were activated for 2 hours at 37°C as described above, and the secretion of TNFα in culture supernatants was evaluated using the Rat TNF ELISA Set (BD Biosciences, San Diego, CA, USA).

### Flow cytometric analysis of Calcium mobilization and membrane FcεRI expression

For the determination of the intracellular free calcium concentration [17], 10^6^ cells were stimulated with anti-DNP IgE for 2–3 hours at 37°C with gentle stirring. Cells were preloaded with 5 µM Fluo-3AM (Molecular Probes, Life Technologies Ltd) in the presence of 0.2% Pluronic F-127 (Molecular Probes) for 30 minutes. Cells were activated by the addition of DNP-KLH at a final concentration of 200 ng/ml and the intracellular free calcium concentration was monitored with a FC500 flow cytometer (Beckman Coulter, Inc. Brea, CA, USA).

For the evaluation of surface expression of FcεRI, cells were incubated for 2 h at 37°C with anti-DNP IgE. The membrane-bound IgE was detected using biotinylated anti-mouse Ig (BioLegend, San Diego, CA, USA) followed by Fitc-conjugated streptavidin (GE Healthcare, Buckinghamshire, UK).

### Production and purification of antibody fragments

Antibody fragments were expressed in E. coli after cloning in pET23NN vector as described [13]. The resulting antibody fragment is tagged with a c-Myc and a 6xHis tag at its C-terminus. For pull-down experiments, antibody fragments were purified from bacterial cytoplasmic extracts using magnetic nickel beads (Ademtech, Pessac, France).

The production of bivalent antibodies used in immunofluorescence experiments was achieved by cloning antibody fragments between BglII and EcoRI site of vector ps1119 [18], allowing the production of N-terminal fusions to a mouse Fc of the IgG1 isotype. 293T cells were transiently transfected using JetPEI (Polyplus, NY, USA) and grown for 6 days. Culture supernatants enriched in antibody-Fc fusion were harvested, filtered on 0.2 µm and stored at −80°C.

### Target pull-down

Cells were incubated overnight at 37°C with anti-DNP IgE and they were activated with 50 ng/ml of DNP-KLH for 3–10 minutes at 37°C as described above. After washing with cold PBS containing phosphatase inhibitors (100 mM sodium fluoride, 5 mM sodium orthovanadate), cells were lysed for 15 minutes on ice with lysis buffer (PBS supplemented with 0.5% sodium deoxycholate, 1% NP-40, 0.1% SDS). Cell lysates were clarified by centrifugation for 15 minutes at 4°C at 16,000 g. The total protein content of the soluble fraction was quantified using the BCA assay kit (Interchim, Montluçon, France).

For pull-down experiments, 3 mg of protein lysates were incubated with 20 µl of magnetic nickel beads loaded with 20 µg of purified antibody fragment (see above) for 2 h at 4°C. Beads were washed 3 times in lysis buffer supplemented with 10 mM imidazole. Elution was performed by the addition of 500 mM imidazole to the beads. Eluates were analyzed by SDS-PAGE.

### SDS-PAGE and MS/MS analysis

Captured proteins were separated by SDS-PAGE (10%) and detected with Coomassie-brilliant blue staining. Bands of interest were cut off from the gel. Gel pieces were subjected to in-gel digestion with trypsin (Promega, Fitchburg, WI, USA) as described [19]. Desalted peptides were resolved on an Ultimate 3000 nano-LC System (Dionex, Thermo Fischer Scientific) equipped with a PepMap 100 C18 column (3 µm particles, 10 nm pore size, 75 mm id ×15 cm). For MALDI MS/MS analysis, column effluent was mixed in a 1/3 ratio with MALDI matrix (2 mg/ml a-cyano-4-hydroxycinnamic acid (LaserBio Labs, CA, USA) in 0.1% TFA, 70% acetonitrile) and spotted on an Opti-TOF 384-well Insert 123×81mm plate. MALDI plates were analyzed using the 4800 Plus MALDI TOF/TOF Proteomics Analyzer mass spectrometer (AB Sciex, MA, USA) in positive reflector ion mode. Each MS spectrum is the result of 1500 averaged laser shots. In MS/MS mode, fragmentation of the 12 most intense selected precursors was performed at collision energy of 2 kV, each MS/MS spectrum is the result of 3000 laser shots. Protein identifications were performed in Uniprot/Swiss-Prot2012_01 database by ProteinPilot Software V 2.0.1 (AB Sciex) using the Paragon algorithm. This software calculates a confidence percentage that reflects the probability that the hit is a false positive, meaning that at 99% confidence level (unused score>2), there is a false positive identification chance of about 1%. After database searching, only proteins identified with an unused score ≥2 and peptides identified with a confidence score ≥95 were retained.

### Western blot

Following SDS-PAGE electrophoresis, proteins were transferred on a 0.2 µm nitrocellulose membrane. Before each hybridization, the membrane was blocked in 5% BSA in TBS-T buffer (10 mM Tris pH 7.4, 150 mM NaCl, and 0.1% Tween) for 1h at room temperature. Proteins were revealed using primary and secondary antibodies coupled to peroxidase according to manufacturer's recommendation. For the signal detection, the ECL-Plus chemiluminescent substrate (PerkinElmer, Waltham, MA, USA) and a camera (G-Box, Syngene, Cambridge, UK) were used. Signal intensities were quantified using the manufacturer-supplied software.

### Immunofluorescence

The cells were seeded on glass slides in Labtek chambers (Nunc, Thermo Fischer Scientific). All stages of these experiments were performed at room temperature. After two washes in PBS, cells were fixed with 3.7% paraformaldehyde (Sigma Aldrich) for 10 minutes, and then permeabilized with PBS containing 0.1% triton X-100 and 1.5% FCS for 10 minutes. Cells were incubated with the primary and the fluorescent antibodies for 1 to 2 h at room temperature. 5H4-VH-Fc fusion protein and a commercial rabbit anti-C12orf4 antibody were detected by Alexa 488 conjugated anti-mouse IgG and Alexa 594 conjugated anti-rabbit IgG antibodies, respectively. After washing, the slides were mounted in Mowiol, then visualized and captured using a Zeiss LSM 510 Meta Confocal Microscope (Oberkochen, Germany).

### shRNA

The two shRNA (sh1: cattctaatctctcggaaa; sh2: agaattgattggcgaaaga) were cloned into vector pSIREN (Clontech, Mountain View, CA, USA). Retroviral supernatants were produced as described above. Infected RBL-2H3 cells were selected by addition of 2.5 µg/ml of puromycin (HyClone, Fischer Thermo Scientific) to the culture medium two days after retroviral infection.

### RT-qPCR

Total RNA was extracted and purified from 10^5^–10^6^ cells using a Qiagen kit. 1 µg of RNA was reverse transcribed using 100 ng of random primer and the M-MuLV Reverse Transcriptase (Invitrogen). qPCR was performed on the cDNA using 2 pairs of primers (ccaaagcgtatgctgagaca/cctgcatcaccttttccatt and ctggaaaccaaaatggagga/cgagcagtgatgtttcctga) and SYBR Green I Master mix on a Light Cycler 480 (Roche, Basel, Switzerland). The data were analyzed using the software supplied by the same manufacturer.

## Results

We performed the phenotypic selection in the rat basophilic leukemia cell line RBL-2H3, used extensively to study FcεRI and the biochemical pathways for secretion in mast cells. First, we sub-cloned a single chain antibody fragment (scFv) library optimized for intracellular expression [13], [20] in plasmid and retroviral eukaryotic expression vectors, both designed for cytoplasmic expression of intrabodies, and two libraries of a diversity of 10^9^ and 2×10^8^ were generated respectively. The recombinant vector pools were subsequently used to transfect the RBL-2H3 cell line in order to generate two distinct populations of 5×10^7^ transformed cells. Previous studies reported an Annexin-V binding assay as a powerful method to monitor mast cell degranulation for functional analysis [21]. The IgE-dependent stimulation of the cells leads to the exposure of exocytosing granules and phosphatidylserines that can be monitored, in proportion to the extent of allergic mediator release, by the binding of exogenously added Annexin-V. We used this method to isolate by flow cytometry cell sorting (FACS) the population of intrabody-containing RBL-2H3 cells that displayed an impaired degranulation.

Both transfection approaches have advantages and drawbacks. Using plasmid expression allows a higher expression level with the inconvenience of several different intrabodies expressed in the same cell. We reasoned that in the case of a dominant phenotype this should not preclude the selection of inhibitory molecules. On the opposite, using retroviral transfection ensured that only few different intrabody genes were present in each cell, presumably allowing a more efficient selection. However, in the latter case the explored diversity was limited to the number of transfected cells (5×10^7^), whereas in the former case the diversity was the product of the cell number by the number of plasmids per cell (2000 copies) and was thus only limited to the size of the initial library (10^9^). Thus, we opted for using both approaches because this would allow not only to explore the limiting factors of each system but also to compare the results obtained from two independent selections.

### Plasmid library selection

**Figure 1:**
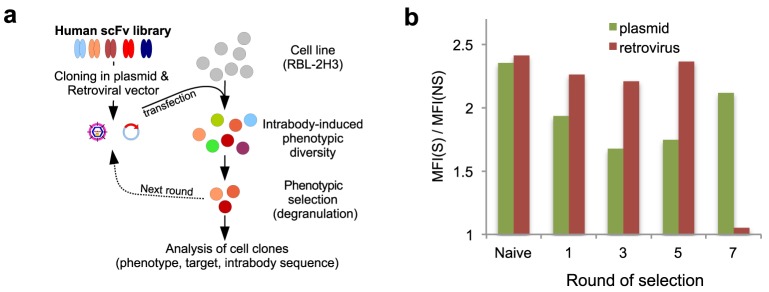
Selection of intrabodies that inhibit mast cell degranulation. a) Schematic view of the selection method. The scFv/intrabody library previously described [13] was cloned in plasmid and retroviral vectors and used to transfect the RBL-2H3 cell line in order to induce a phenotypic diversity in a collection of cells. Clones displaying the desired phenotype, measured by inhibition of degranulation, were selected and the couple constituted by the inhibitory intrabody and its target antigen was identified and characterized. b) Annexin-V staining of cell populations from the library selection rounds is illustrated as the ratio of the geometric mean (MFI) of the FcεRI-stimulated (S) to the unstimulated (NS) cells (Fig. S1).

In the case of the plasmid library, seven rounds of enrichment were performed based on a selection method comprising the following steps: a) FcεRI-mediated cell stimulation followed by Annexin V-APC staining; b) FACS sorting of 10% of the population corresponding to the less fluorescent cells; c) extraction of the plasmid DNA pool; d) amplification of the scFv genes and their subsequent cloning in the same expression vector (Fig. 1a). This procedure allowed the enrichment for cells containing intrabodies able to block cell degranulation as shown by the decrease of the Annexin V staining of stimulated versus unstimulated cells (Fig. 1b, Fig. S1a). In addition, because each round of selection included a recloning step of scFv genes, this procedure ensured that the observed phenotype was specifically due to the transfected intrabody genes and not to a cell drift. Another issue with the selection was the possible anti-apoptotic effect of monomeric IgE used for FcεRI stimulation [22], [23]. However, since we have incubated both the unstimulated and the stimulated cells with IgE before the addition of the antigen DNP, if present this effect should be identical in both populations and should not have influenced the selection.

**Figure 2:**
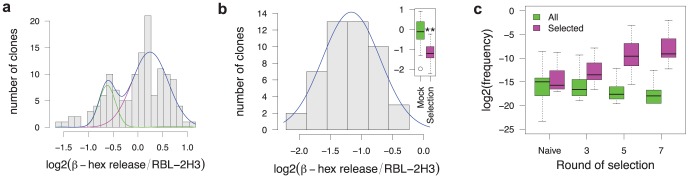
Analysis of selected clones. a) Distribution of the β-hexosaminidase release measured on 126 stable clones isolated from the last round of the plasmid selection. The distribution profile does not fit a normal distribution (p = 0.027 using Jarque-Bera Normality Test) and is skewed to the left (p = 0.009 using Agostino's skewness test [53]). The blue curve is the sum of the two normal distributions plotted in magenta and green and was fitted to the distribution. b) Distribution of the β-hexosaminidase release measured on 48 retroviral clones. The distribution is normal (p = 0.92) and the blue curve is the best normal distribution fitted to the data. Inset: boxplot of the β-hexosaminidase release of 48 retroviral clones compared to 11 mock-transfected RBL-3H2 clones. **: p<0.01 (Student t-test). Boxplot whiskers extend to the most extreme data point that is no more than 1.5 times the interquartile range. c) VH sequence evolution during retroviral selection followed by high throughput sequencing. All: frequency of the 6789 VH sequences present in the four sequenced pools. Selected: enrichment of the 125 DNA sequences (62 different CDR3 in amino acid) forming the 10 selected families (Fig. S4 & S5). Boxplot whiskers extend to the most extreme data point.

To identify individual intrabody sequences responsible for the inhibitory phenotype, we generated stable RBL-2H3 clones from the pool of plasmids obtained from the seventh round of selection. Using qPCR, we estimated that during the selection, each cell contained an average of 2,000 plasmids, thus we reasoned that most of the cells should contain non-inhibitory passenger intrabodies. One hundred and twenty-six stable clones were tested in a degranulation assay based on the measurement of the FcεRI-mediated release of the enzyme β-hexosaminidase. As shown in Fig. 2a, the β-hexosaminidase release values are distributed following a bimodal distribution with a major peak at 110% and a smaller one at 65%. The former peak contains cells expressing non-inhibitory intrabodies whereas the latter corresponds to the low-degranulating clones that express inhibitory intrabodies and which represent about 20% of the clones. The analysis of the intrabody sequences expressed in 36 clones revealed a high diversity with 1–2 different sequences expressed in each clone (Fig. S2).

### Retroviral library selection

In the case of the retroviral library, the screen was performed in the same manner. Since retroviral infection directly generated stable clones, extraction of the intrabody genes and recloning between each round was not necessary. We however introduced a recloning step after the third and the fifth round of selection. This ensured that the retrovirus-induced phenotype was associated with the expressed intrabody sequence and was not due to a cell drift or a particular genomic insertion site. Inhibition of the phenotype was clear after seven rounds of selection as the shift in Annexin V of FcεRI-stimulated cells was totally abolished at round 7 (Fig. 1b & Fig. S1b). An aliquot of cells from selection round 7 was seeded at limiting dilution and 48 isolated clones were analyzed in triplicate by measuring the release of β-hexosaminidase (Fig. 2b). Contrary to the plasmid selection, the distribution of the β-hexosaminidase release values was monomodal and significantly inhibited by 54% on average when compared to 11 mock-transfected clones. This showed that the retroviral library selection was more powerful than the plasmid one, presumably because of the presence of fewer intrabodies per cell, which reduced the co-selection of passenger intrabodies to a minimal.

We used high throughput sequencing for the analysis of the intrabody diversity evolution in the cell population during the course of selection and for the identification of the best inhibitory intrabodies. This avoided a biased analysis of a limited number of cellular clones that may bear peculiar properties. scFv sequences expressed in the initial pool of RBL-2H3 infected cells before the first selection (naive library), and from cells from each of rounds 3, 5, 7 and 8 were analyzed. Sequencing of 10^6^ clones from the naive library revealed that 80% of the sequences all had H3 or L3 without frameshift or stop codon. The translation of the nucleotide sequences generated 250,000 unique VH amino acid sequences and 350,000 VL sequences. This reflects the actual diversity of the library contained in 10^6^ cells but do not take into account the additional diversity due to the random pairing of VH and VL chains (Fig. S3).

Since the VH domain is known to be the most important determinant of antibody affinity and specificity [24], we only analyzed the CDR3 sequences that correspond to the variable part of the VH domains in our library design [13]. By setting the False Discovery Rate to 0.05, 2568 VH DNA sequences appeared to be enriched and none significantly depleted during the course of the selection using the SAMSeq software [15]. The fact that no sequence declined significantly during the selection shows that none of the intrabodies was toxic enough to the cell to induce an early apoptotic phenotype and to be selected. Enriched sequences were translated and we retained the 108 VH sequences that continuously increased during the course of the selection. These 108 VH represented 40% of the sequences present in the final selected library and were grouped in 69 families using unsupervised hierarchical clustering and a cutoff of 60% identity (Fig. S4). Thirty-nine families contained a unique sequence and were not further analyzed. The remaining 69 VH clustered in 17 groups, and we retained the 10 VH families present at least at 0.1% (0.1–25%) in the final enriched library. Between the naive library and the final selected library, each of these 10 families was enriched about 150 fold (90–215), however some individual sequences were enriched more than 500-fold. In addition, whereas the frequency of most of the clones present in the initial library decreased at an exponential rate during the course of the selection, the 10 identified families were strongly enriched (Fig. 2c & Fig. S5).

### Characterization of individual clones

**Figure 3:**
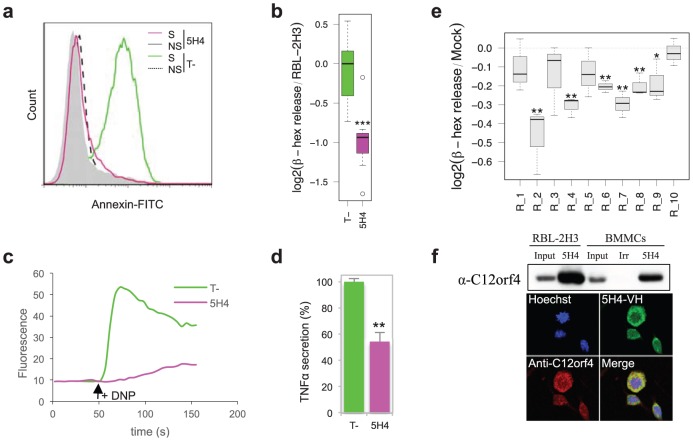
Anti-C12orf4 intrabody inhibits mast cell degranulation. Analysis of stable clone 5H4: a) measurement of Annexin-V staining; b) β-hexosaminidase release; c) calcium flux and d) TNFα secretion. T-: Irrelevant intrabody. S: IgE/DNP stimulated. NS: unstimulated. Boxplot whiskers extend to the most extreme data point that is no more than 1.5 times the interquartile range. e) Measure of β-hexosaminidase release by retroviral infected populations. Clone R_8 is identical to the intrabody expressed by the plasmid clone 5H4. Sequences of the clones are given in Fig. S6. Boxplot whiskers extend to the most extreme data point. f) Specific binding of 5H4-VH to C12orf4. Top panel: pull-down assay using 5H4-VH as capture agent and a commercial anti-C12orf4 polyclonal serum to reveal the protein. Irr: Irrelevant VH fragment, differing from 5H4 VH only by its CDR3 sequence. Low panel: subcellular localization of C12orf4 analyzed by confocal laser microscopy after double staining. Top left: Hoechst; top right: 5H4-VH-Fc fusion; bottom left: anti-C12orf4 commercial antibody; bottom right: merge. *: p<0.05; **: p<0.01; ***: p<0.001 (Student t-test).

Next, 136 unique VH sequences obtained from 178 clones of the plasmid library (stable clones and randomly picked sequences from the last round of selection) were compared with the 108 VH sequences enriched during the retroviral selection. Only one VH sequence corresponding to clone 5H4 was found common to both selections. Moreover, 5H4 family (R_8) is part of the 10 best families selected from the retroviral sequence analysis and the 5H4-VH sequence was enriched 170 fold during the retroviral selection. Plasmid-derived stable RBL-2H3 clone 5H4 did not show any Annexin-V staining following FcεRI stimulation (Fig. 3a) and displayed strong defects in β-hexosaminidase release, calcium flux and TNFα secretion (Fig. 3b, 3c, 3d). Analysis of 5H4 sequence revealed that the gene encoding scFv 5H4 was truncated at its C-terminus because of the presence of a stop codon in the first codon of the light chain CDR3. Since the original scFv library contained only variable CDR3 loops, we reasoned that the inhibitory phenotype of 5H4 was carried by its VH portion. Indeed, isolated human VH domains have already been shown to be efficient intrabodies, particularly when the VH sequence belongs to the human VH3 family as it is the case here [25], [26]. In fact, the analysis of the FcεRI-mediated degranulation of the retroviral clone R_8 confirmed the inhibitory potential of the intrabody 5H4-VH (Fig. 3e).

We also evaluated the inhibitory phenotype of the 9 other families of intrabodies selected during the retroviral selection. The most frequent sequence of each family (Fig. S6) was cloned and the cellular phenotype of retrovirus-infected mixed cell populations was analyzed. As shown in Fig. 3e, 6 out of 10 intrabodies induced a significant inhibition of degranulation following FcεRI stimulation. These results confirmed that our statistical analysis of the data generated by high throughput sequencing successfully led to the identification of inhibitory intrabody sequences.

### Target identification

The strength of the IBPheS approach lies in the fact that intrabodies are antibody molecules bearing high affinity and specificity which can be used for the identification of their target. In order to identify the cellular target of intrabody 5H4, the gene encoding 5H4-VH was expressed in *E. coli* cytoplasm, and the purified antibody fragment was used to capture its target from RBL-2H3 extracts. The captured proteins were analyzed by mass spectrometry using an irrelevant VH fragment as a control. A unique protein called RGD1311164, the homolog of human protein C12orf4, was identified in three independent experiments with the best score. For an easier reading we will refer to RGD1311164 as C12orf4. The specific binding of 5H4-VH to C12orf4 from rat and mouse origin was confirmed in pull-down experiments performed respectively on RBL-2H3 cell line and murine bone marrow-derived mast cells using a commercial polyclonal serum (Fig. 3f & Fig. S7a). Because 5H4 VH differs from the irrelevant VH fragment only by its CDR3 sequence (10 amino acids), its specificity for C12orf4 is driven by its CDR3 sequence and cannot be due to the exposed hydrophobic VH-VL interface. Immunoprecipitation experiments as well as immunofluorescence analysis using confocal microscopy with either 5H4-VH-Fc or a commercial anti-C12orf4 antibody showed that C12orf4 is a cytoplasmic protein, and that its subcellular localization and expression level does not change upon FcεRI stimulation (Fig. 3f & Fig. S7b).

### Characterization of RGD1311164/C12orf4

We further characterized the role of C12orf4 in FcεRI-mediated mast cell responses. For this purpose, the short hairpin RNA (shRNA) approach was used for down regulation and modulation of C12orf4 expression in RBL-3H2 cells. As shown in Fig. S8a, the two shRNA decreased the mRNA level by 60–80% and the protein level by 55–70%. The analysis of the degranulation of shRNA transfected cells following FcεRI stimulation showed a decrease in β-hexosaminidase release and TNFα secretion that correlated with an inhibited calcium flux (Fig. S8b). These results confirmed using an independent approach that inhibition of C12orf4 leads to a defect in mast cell activation.

**Figure 4:**
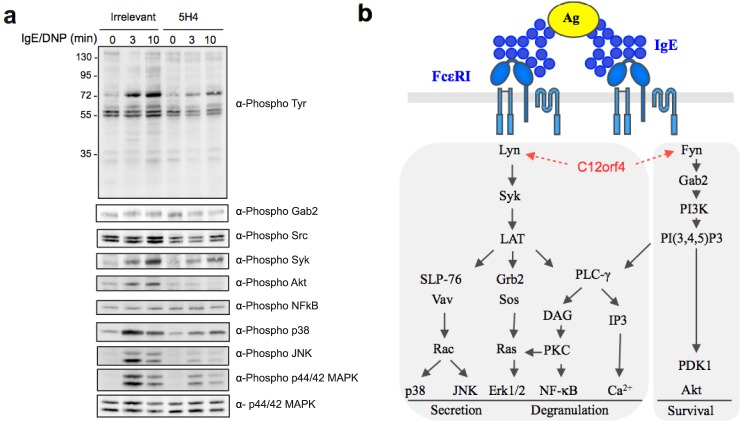
C12orf4 is implicated in the early events of the degranulation pathway. a) Western blot analysis of the FcεRI-mediated phosphorylation of major proteins implicated in mast cell activation. 5H4 expressing RBL-2H3 cells are compared to cells expressing an irrelevant antibody fragment. Cells are either non-activated or activated with IgE/DNP for 3 and 10 minutes as indicated. b) Schematic view of mast cell signaling pathways, kindly provided by Dr. Marc Daëron.

In mast cells and basophils, the engagement of FcεRI initiates the activation of the Src kinases Lyn and Fyn and the Syk kinase, which allows signal propagation through the phosphorylation and activation of several downstream proteins [27]. Lyn phosphorylates Syk that coordinates further signals such as the activation of PLC-γ and calcium mobilization. Fyn initiates a complementary signaling pathway through the adapter Gab2 that is essential for the activation of PI3K and cell degranulation (Fig. 4b). We investigated the impact of targeting C12orf4 on FcεRI-mediated signaling events. Protein extracts of non-stimulated and FcεRI-stimulated stable clone 5H4 were analyzed by western blot using anti-phosphotyrosine monoclonal antibody 4G10. A defect in the FcεRI-mediated tyrosine phosphorylation of proteins migrating at 40–42, 55–60 and 72 kDa was observed. Using antibodies specific to key proteins involved in the signaling pathways, we confirmed a decreased phosphorylation of Src and Syk tyrosine kinases as well as the downstream proteins MAPKs and Akt, demonstrating an impairment of both Lyn- and Fyn-dependent signals (Fig. 4a). These results are consistent with the defect in the degranulation events (Fig. 3b, 3d). The analysis of the protein extracts of cells transfected with a C12orf4-targeting shRNA showed a milder overall effect, with a selective modulation of the Fyn pathway as shown by the decreased phosphorylation of downstream proteins Gab2 and Akt (Fig. S8c). This may be due to the moderate shRNA-mediated inhibition of C12orf4 expression (about 50%). Taken together, our results suggest that C12orf4 plays a role in the early signaling events following FcεRI-induced cell activation.

## Discussion

In this study, we report the identification of protein RGD1311164/C12orf4 as a new player in FcεRI-induced cell degranulation. Database interrogation revealed that C12orf4 is a protein of unknown function. In human, C12orf4 gene is localized on chromosome 12p13.3. Sequence analysis showed that C12orf4 is widely conserved from nematodes to humans, with for instance 94% amino acid identity between rat and human protein sequences. This suggests a common role in all these organisms, but none of the proteins of this family have a defined function or homology to a domain of known function. Genini and co-workers [28] suggested a possible link between C12orf4 gene and the autosomal recessive disease known as arthrogryposis multiplex congenita (AMC), one of the most common congenital defects observed in pigs and in other mammals.

Our data suggest that C12orf4 is a cytoplasmic protein and that its cellular localization is not affected following FcεRI stimulation. RBL-2H3 stable clones expressing anti-C12orf4 5H4 inhibitory intrabody, as well as cells expressing a shRNA against C12orf4, showed an inhibition of mast cell activation and a decrease in allergic mediator release. These cellular responses correlate with defects in the early signaling events following FcεRI stimulation, as shown by a decrease in the phosphorylation of key upstream proteins such as Src kinases. Because the level of FcεRI expression at the membrane of transfected cells was not affected, C12orf4 is presumably acting upstream of Src kinases, but is not implicated in receptor recycling and degradation. We analyzed 5H4-pull down experiments with antibodies specific to FcεRI, Lyn, Syk and with the anti-phosphotyrosine monoclonal antibody 4G10, and none of the corresponding targets were detected. This is not uncommon since some signaling molecules interact weakly with their partners and it is sometimes difficult to demonstrate co-immunoprecipitation of endogenous proteins. Another hypothesis is that 5H4 may inhibit the interaction of C12orf4 with its cellular partners, precluding detection of interacting proteins in pull-down experiments.

The IBPheS method described here allowed us to identify several other intrabodies that affect the degranulation of RBL-2H3 cells. Presumably, some of these intrabodies recognize known targets implicated in the degranulation process. However, the diversity of the used intrabody repertoire is somewhat limited because of the difficulty to manipulate more than 10^8^ cells, and thus the approach cannot allow a complete enumeration of all the players implicated in the studied phenotype. This however demonstrates that the selection of intrabodies as phenotype-modulators can be accomplished in cell-based assays using unbiaised combinatorial libraries (not preselected by phage panning) and without any previous knowledge of a target. On this line, two recent studies describe the use of large and naïve intracellular combinatorial libraries for phenotypic modulation. The first study reports the selection of secreted integrin-binding agonist antibodies that convert stem cells to dendritic cells [29]. The second approach describes the use of lentiviral libraries of intrabodies for the selection of scFv fragments that block rhinovirus-induced cell death [30]. In this case, the studied phenotype is highly selectable because based on cell survival. This explains the very impressive enrichment factor of more than one million-fold since only the cells containing protective intrabodies survived viral infection. In the present work, we show that despite a weaker FACS-based phenotype, the selection is efficient enough to isolate inhibitory intrabodies. This makes the approach applicable to a larger number of cellular phenotypes. Indeed, when coupled with high throughput sequencing, the method is able to identify very rare clones. In fact, the sequence analysis revealed that intrabodies that were present at a frequency of 10^−7^ in the naive library were significantly enriched and identified in the 10 families of retroviral clones (Fig. S5). Thus, as in the case of phage-display selection, the use of high throughput sequencing allowed the identification of clones even expressed well below the background and impossible to select using a direct cloning strategy [31].

If we compare IBPheS to classical genetics, the intrabody plays the same role than mutations. However, because the intrabody does not directly modify its target but modulates its function, it is also analogous to a chemical drug. Thus the system described here has the advantages of both approaches, that is the power and flexibility of genetics coupled with the clinical applications of pharmacology. Intrabodies have demonstrated they are able to recapitulate all the antibody properties within the cell: enzyme inhibition [32]; breaking protein-protein [33] and protein-DNA interactions [34]; re-activating mutant enzymes [35]; targeting specific protein conformations [36], [37], domains [17], and post-translational modifications [38]; and inducing protein degradation [39], [40]. In addition, by targeting them to specific cell compartments intrabodies can re- or de-localize their target [41] and block secretion [42]. Intrabodies are thus able to mimic the whole spectrum of mutations that can be obtained in classical genetics. However, in addition to proteins, antibodies are also able to recognize small chemicals, usually referred as haptens, glycans and lipids [43], [44]. As such they represent a powerful tool not only to interrogate the proteome diversity but also secondary messengers and metabolism in cells. As such the IBPheS method must be seen as a complement to other methods such as genome-wide shRNA screens [45]–[47], but with specific advantages associated with the direct targeting of effector proteins instead of mRNA.

Intrabodies have also proven their potential to be used in clinics [48]. Delivery of an anti-erbB2 intrabody using an adenovirus vector has been described in a phase I clinical trial with minimal toxicity [49]. The main current limitation is intrabody gene delivery but this will improve with advances in gene transfer technology. An alternative approach still not demonstrated in the case of intrabodies could be the use of internalizing peptides or liposomal vehicles to directly deliver proteins within tissues in pre-clinical models and in patients [50]. However, since this has already been described in cell cultures [51], [52], this route of intrabody delivery could be a viable solution. As an easier and more generally applicable solution, we reported the use of an antibody displacement assay to convert an intrabody directed against the tyrosine kinase Syk into chemical drugs [7], [8]. The isolated molecules recapitulated the intrabody effects in cell cultures and were able to block the anaphylactic shock when administrated orally in animal models [7].

In conclusion, the IBPheS method aims to be an integrated approach for the concomitant identification of a protein target and an intrabody as a lead inhibitor. As such, and compared to other large-scale approaches, this represents a straightforward path to the discovery of potential therapeutic molecules.

## Supporting Information

Figure S1 **FACS analysis of the IgE/DNP stimulated (S: green) and non-stimulated (NS: black) RBL-2H3 cells transformed with plasmid (a) or retroviral (b) libraries.** Naive: unselected library; Round n: enriched library after n rounds of selection. The X axis represents Annexin V labelling, and the Y axis the Forward Scatter (FCS).
(PDF)

Figure S2 **Individual clone phenotypes from plasmid library selection.** a) 133 stable clones were tested for β-hexosaminidase release. Red dots represent the clones selected for sequencing. The clone 5H4, characterized in Fig. 3 of the manuscript, is marked. b) 36 clones were sequenced. Clones are sorted from the least to the most degranulating clone in the initial screen in (a). Nb seq: number of intrabody sequences retrieved by PCR. “> = 2”: the clone contains more than 1 sequence but was not analyzed further to determine the exact number of inserted intrabody. H3: VH CDR3 sequence. L3: VL CDR3 sequence. *: stop codon. x: unread because of poor sequencing quality. c) Clones for which a sequence was determined, were re-tested for β-hexosaminidase release (between 2 and 6 replicates). *: p<0.05; **: p<0.01; ***: p<0.001 (Student t-test). Clones 6E8, 8D12 and 5F5 were used as negative controls.
(PDF)

Figure S3 **Intrabody library diversity.** Sequence analysis of DNA extracted from one million clones infected with the indicated retroviral libraries. Nb of reads: number of reads for each library; Nb of seq (dna): Number of different full length CDR3 DNA sequence; Nb of seq (dna no stop): Number of different full length CDR3 DNA sequence without stop codon or frameshift; Nb of seq (aa): Number of different CDR3 protein sequences obtained without stop codon or frameshift. a) VH CDR3. b) VL CDR3. Round3a and Round3 are the same pool but sequenced before and after recloning respectively (see Materials and Methods).
(PDF)

Figure S4 **Clustering of VH sequences identified in retroviral library selection.** Sequence analysis of the 108 VH enriched during retroviral selection. Sequences are aligned according to IMGT numbering scheme. The two numbers at the left of each sequence are the frequency in the selected library (round 7) and the enrichment factor between naive and final library, respectively. The 10 retained families after clustering are indicated.
(PDF)

Figure S5 **Population evolution of the clones from the 10 selected families.** For each family in Fig. S4, the evolution of the frequency of all the clones is plotted. The sequence above the plots is the VH CDR3 sequence of the most abundant clone used in the validation study in Fig. 3e. Since clones were considered as different when their DNA sequences were different, the number of clones in each family does not necessary match the number in Fig. S4 that compared translated CDR3 sequences.
(PDF)

Figure S6 **Sequences of the most abundant retroviral clones from the 10 selected families.** R_7 has a stop codon in the VL CDR3 loop and is thus truncated and expressed without the C-terminal eGFP tag. R_8 has the same VH than plasmid clone 5H4 and has been cloned as a single VH domain in retroviral vector.
(PDF)

Figure S7 **Analysis of FcεRI-induced C12orf4 expression and subcellular localization.** a) Pull-down assays were performed on total lysates of non-stimulated and FcεRI-stimulated RBL-2H3 cells, using 5H4-VH and an irrelevant VH fragment. The presence of C12orf4 in protein extracts and pull-down fractions (PD) was detected using a rabbit anti-C12orf4 polyclonal serum. b) Analysis of subcellular localization of C12orf4 following FcεRI-stimulation. RBL-2H3 cells were either non stimulated (top panels) or stimulated for 3 minutes (middle panels) and 10 minutes (bottom panels) with IgE/DNP as described in methods and stained with a commercial rabbit anti-C12orf4 serum followed by an anti-rabbit IgG Alexa 599 labeled secondary antibody (left panels). Nuclei were stained with Hoechst (right panels).
(PDF)

Figure S8 **shRNA-induced down-regulation of C12orf4 expression.** a) Two shRNA against rat C12orf4 (sh1 and sh2) were cloned in a retroviral vector and transduced in RBL-2H3 cells. Analysis of C12orf4 expression by qPCR and western blot were performed 10 and 15 days post-infection. b) Analysis of β-hexosaminisase release (left), calcium flux (middle), and TNFα secretion (right) were performed with cell populations 5 days post-infection. c) Western blot analysis of the FcεRI-mediated phosphorylation of major proteins implicated in mast cell activation. Cell populations transfected with sh1 C12orf4 (5 days post-infection) are compared with a control shRNA (shLUC), either non activated or activated with IgE/DNP for 3 and 10 minutes. **: p<0.01; ***: p<0.001 (t-test).
(PDF)
