# Supplementary figures and images for: In-Cell Intrabody Selection from a Diverse Human Library Identifies C12orf4 Protein as a New Player in Rodent Mast Cell Degranulation
Source: PLoS One. 2014 Aug 14;9(8):e104998. doi: 10.1371/journal.pone.0104998 (PMC4133367; doi:10.1371/journal.pone.0104998)

**a**

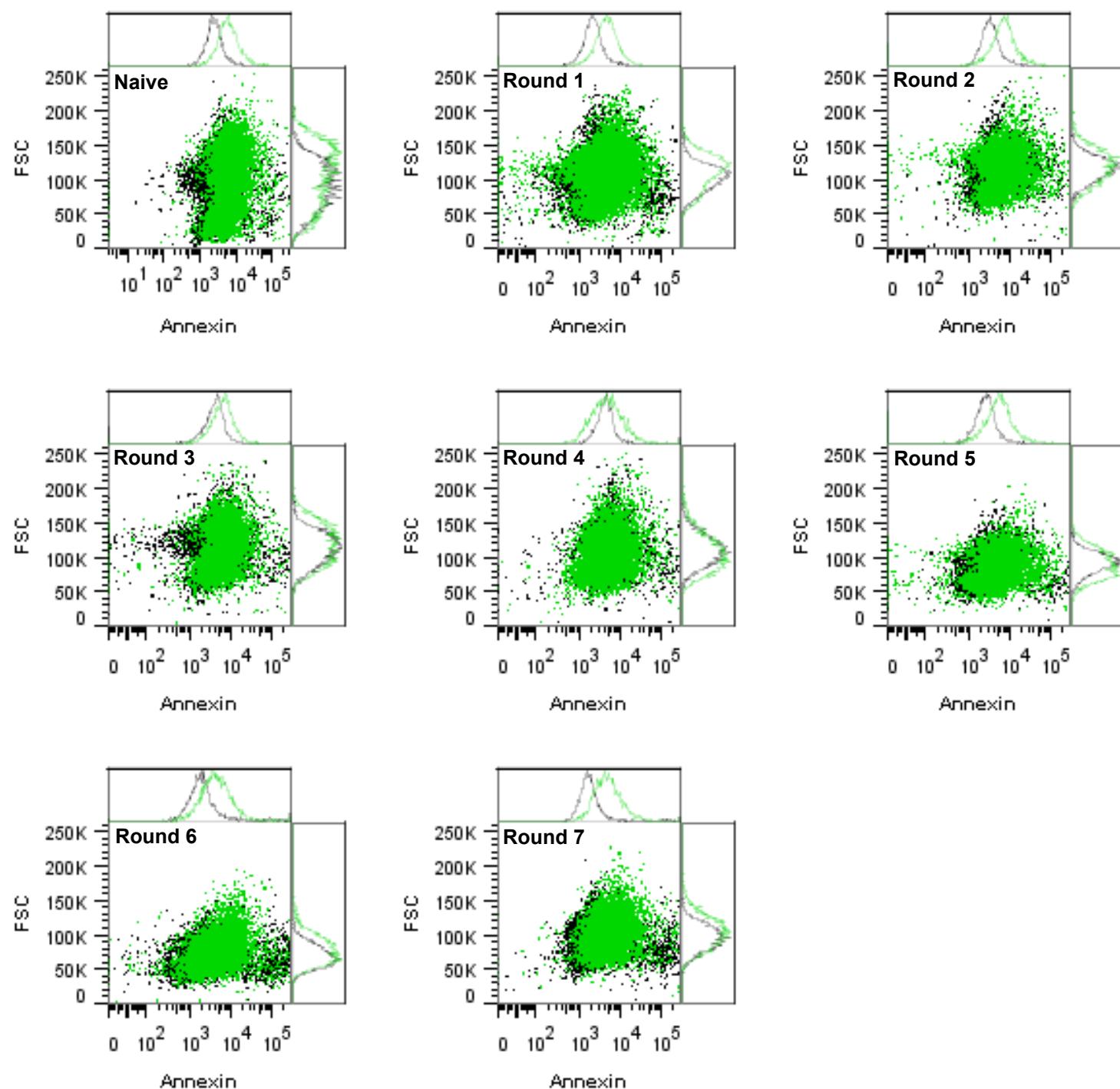

**b**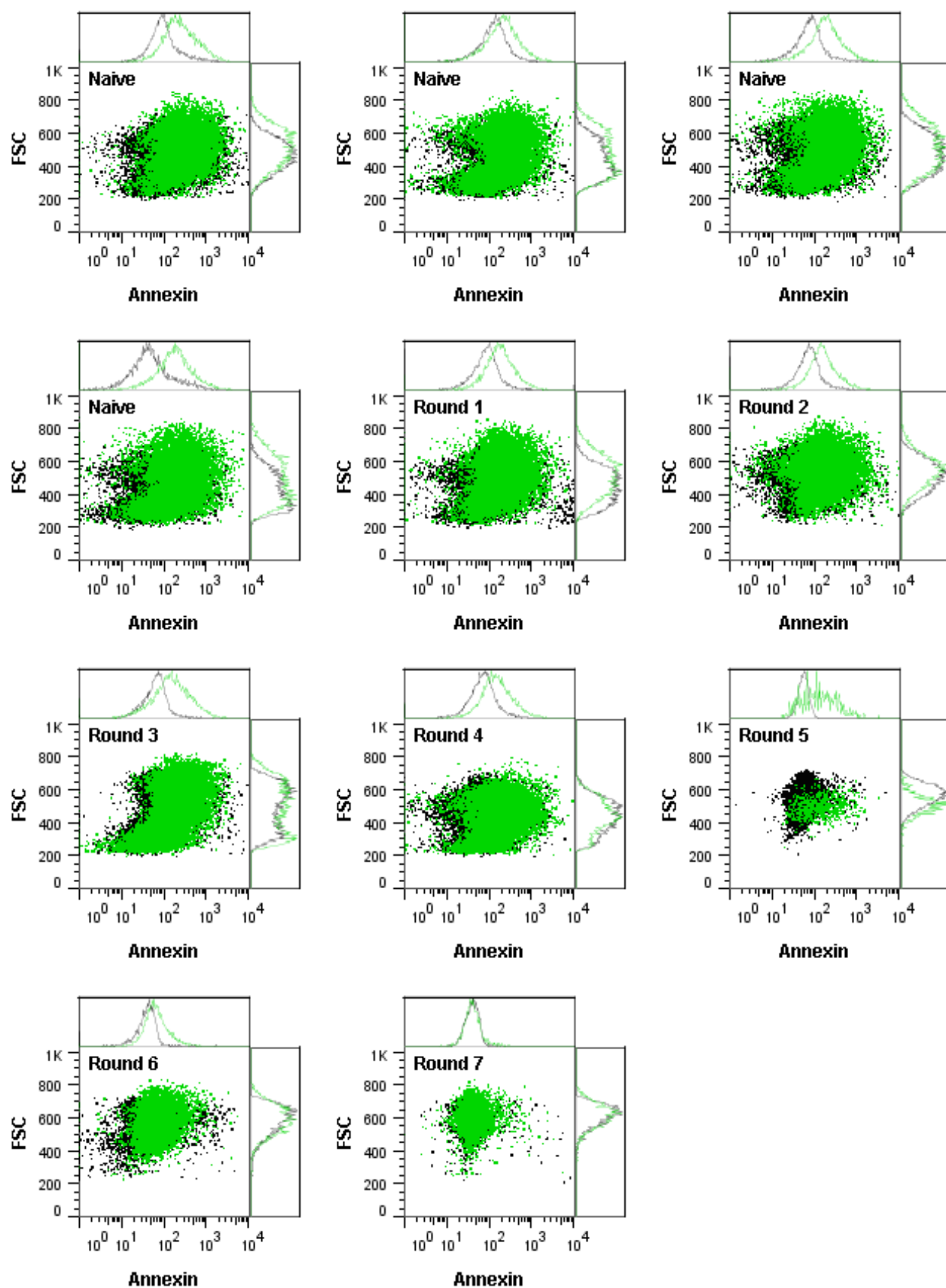

Supplement: Figure S1 — FACS analysis of the IgE/DNP stimulated (S: green) and non-stimulated (NS: black) RBL-2H3 cells transformed with plasmid (a) or retroviral (b) libraries. Naive: unselected library; Round n: enriched library after n rounds of selection. The X axis represents Annexin V labelling, and the Y axis the Forward Scatter (FCS). (PDF) [file pone.0104998.s001.pdf]

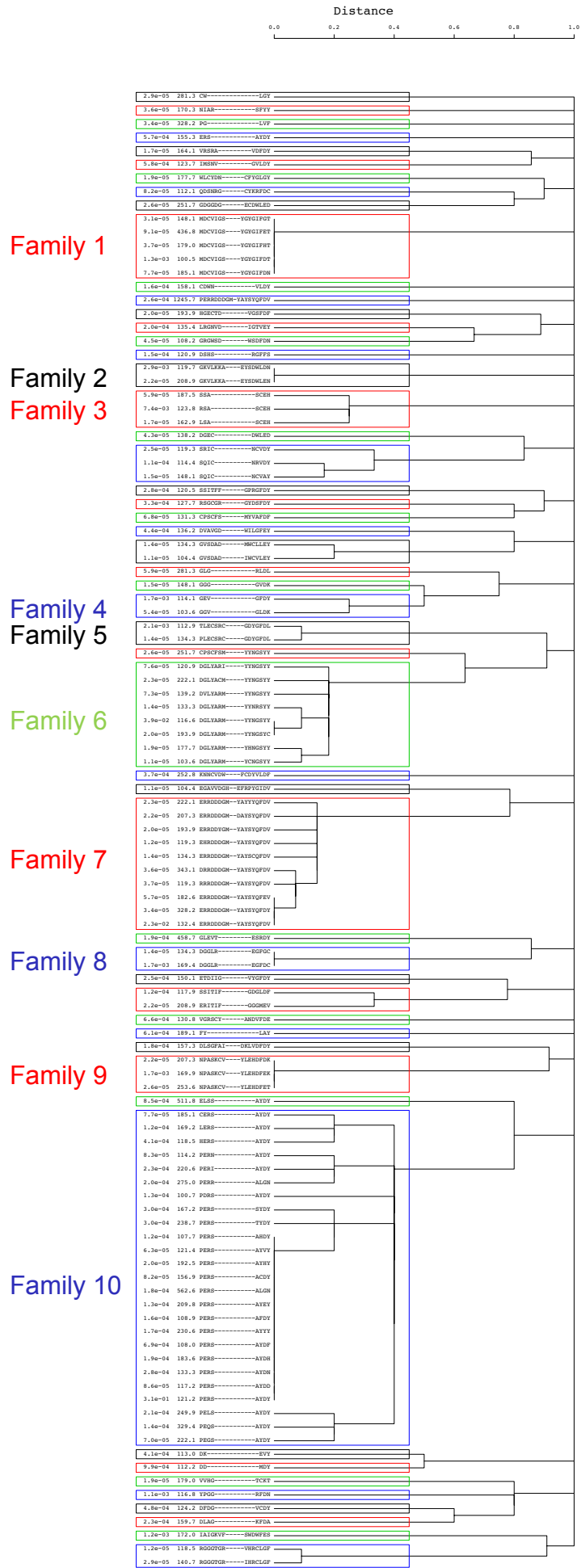

Supplement: Figure S4 — Clustering of VH sequences identified in retroviral library selection. Sequence analysis of the 108 VH enriched during retroviral selection. Sequences are aligned according to IMGT numbering scheme. The two numbers at the left of each sequence are the frequency in the selected library (round 7) and the enrichment factor between naive and final library, respectively. The 10 retained families after clustering are indicated. (PDF) [file pone.0104998.s004.pdf]

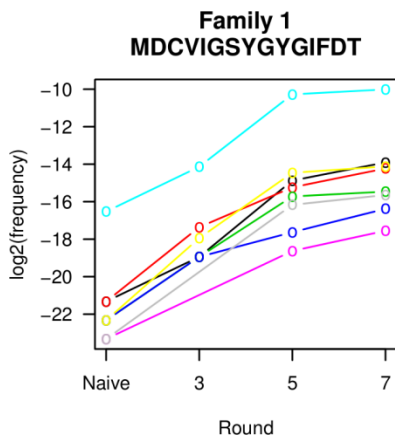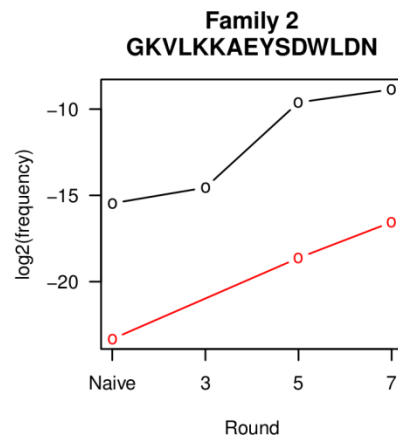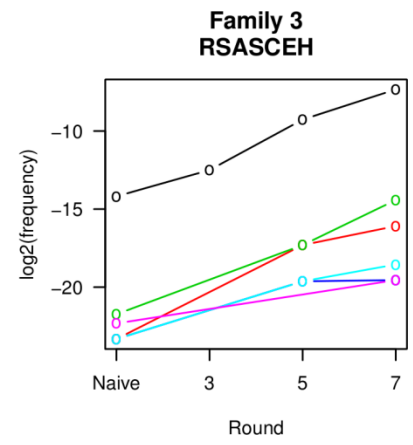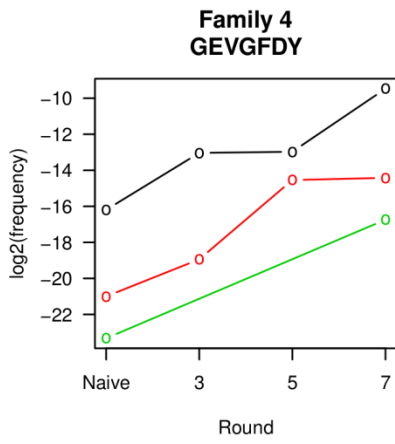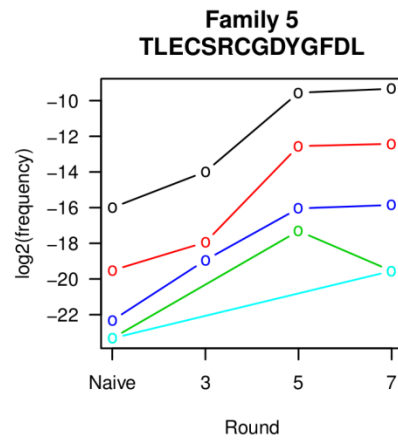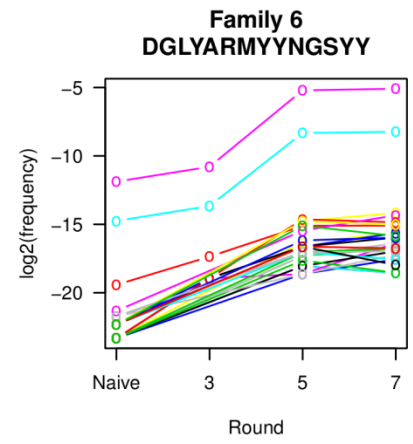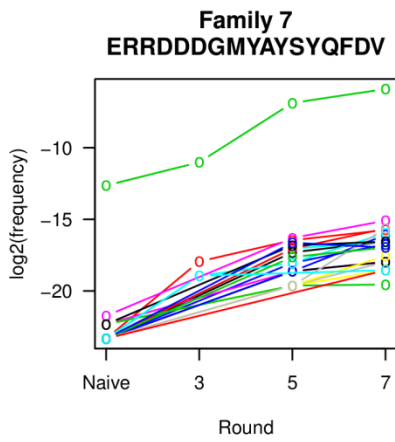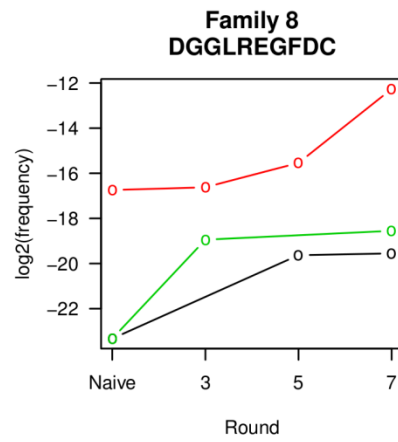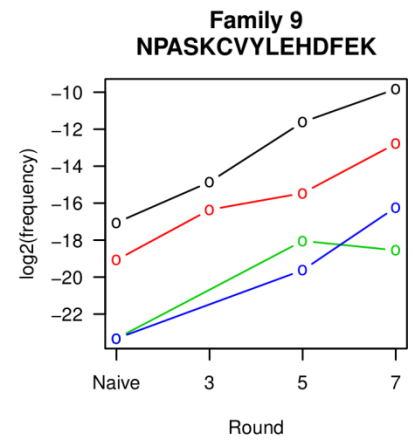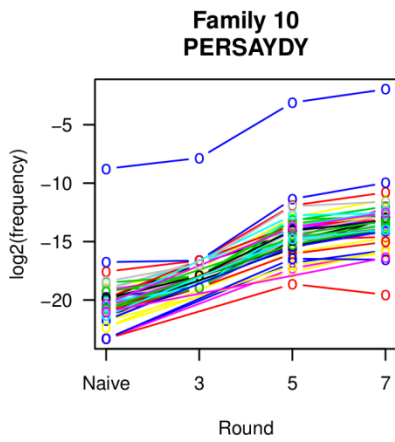

Supplement: Figure S5 — Population evolution of the clones from the 10 selected families. For each family in Fig. S4, the evolution of the frequency of all the clones is plotted. The sequence above the plots is the VH CDR3 sequence of the most abundant clone used in the validation study in Fig. 3e. Since clones were considered as different when their DNA sequences were different, the number of clones in each family does not necessary match the number in Fig. S4 that compared translated CDR3 sequences. (PDF) [file pone.0104998.s005.pdf]

**a**

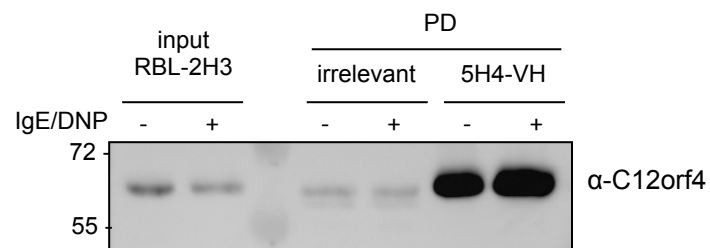

**b**

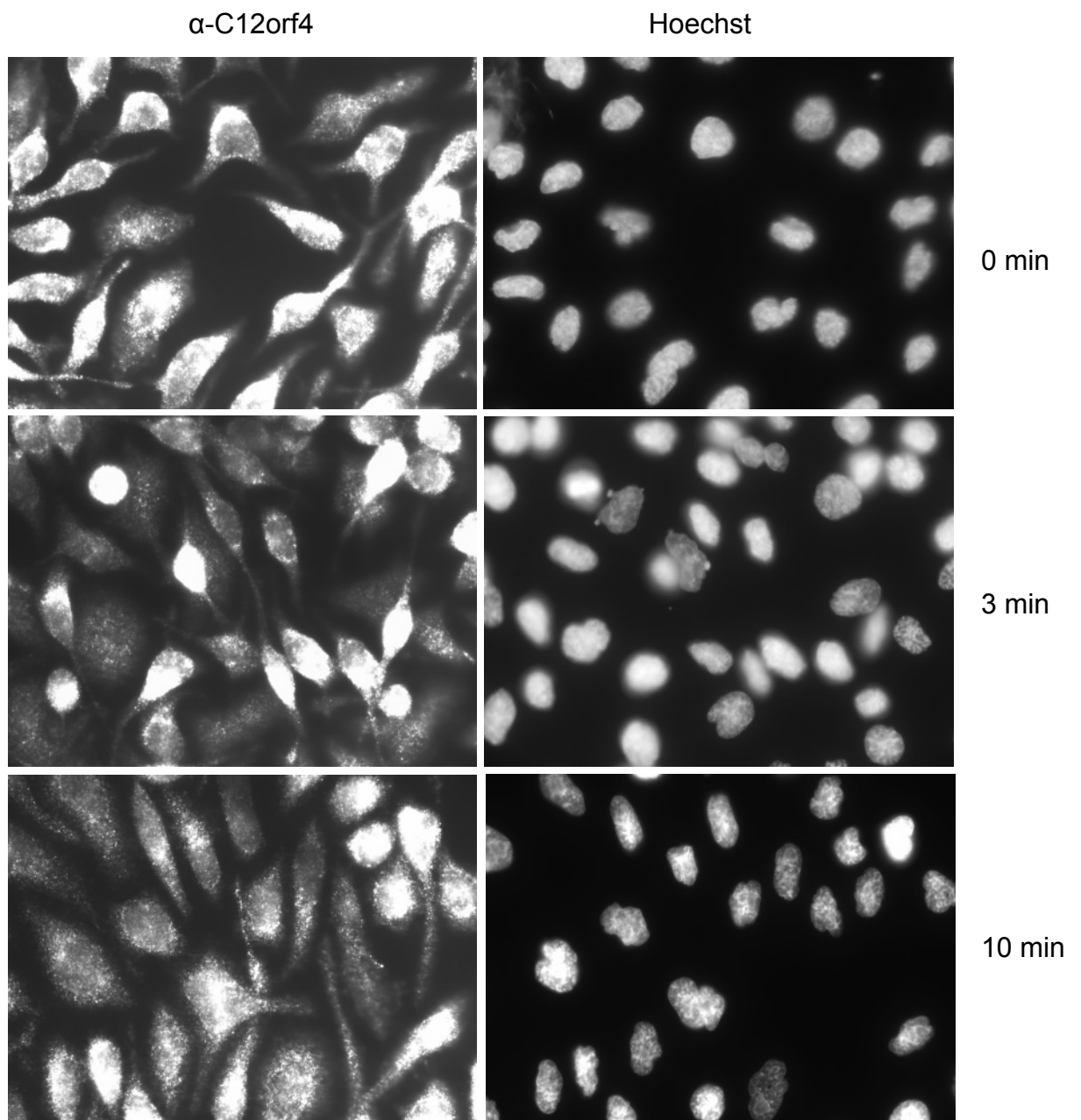

Supplement: Figure S7 — Analysis of FcεRI-induced C12orf4 expression and subcellular localization. a) Pull-down assays were performed on total lysates of non-stimulated and FcεRI-stimulated RBL-2H3 cells, using 5H4-VH and an irrelevant VH fragment. The presence of C12orf4 in protein extracts and pull-down fractions (PD) was detected using a rabbit anti-C12orf4 polyclonal serum. b) Analysis of subcellular localization of C12orf4 following FcεRI-stimulation. RBL-2H3 cells were either non stimulated (top panels) or stimulated for 3 minutes (middle panels) and 10 minutes (bottom panels) with IgE/DNP as described in methods and stained with a commercial rabbit anti-C12orf4 serum followed by an anti-rabbit IgG Alexa 599 labeled secondary antibody (left panels). Nuclei were stained with Hoechst (right panels). (PDF) [file pone.0104998.s007.pdf]

**a**

sh1 CATTCTAATCTCTCGGAAA  
 sh2 AGAATTGATTGGCGAAAGA

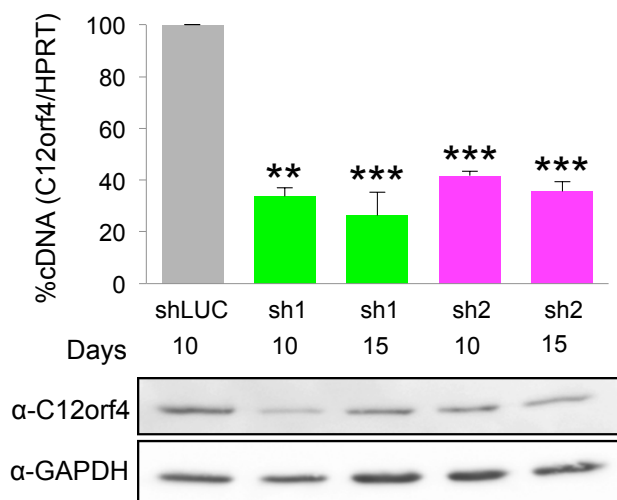**c**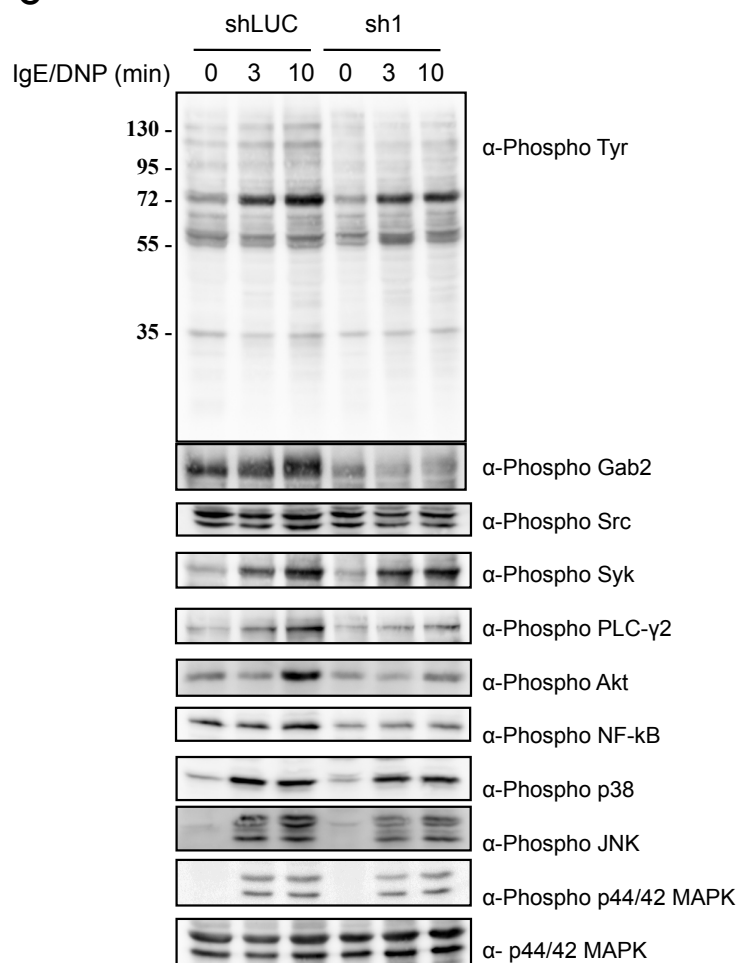**b**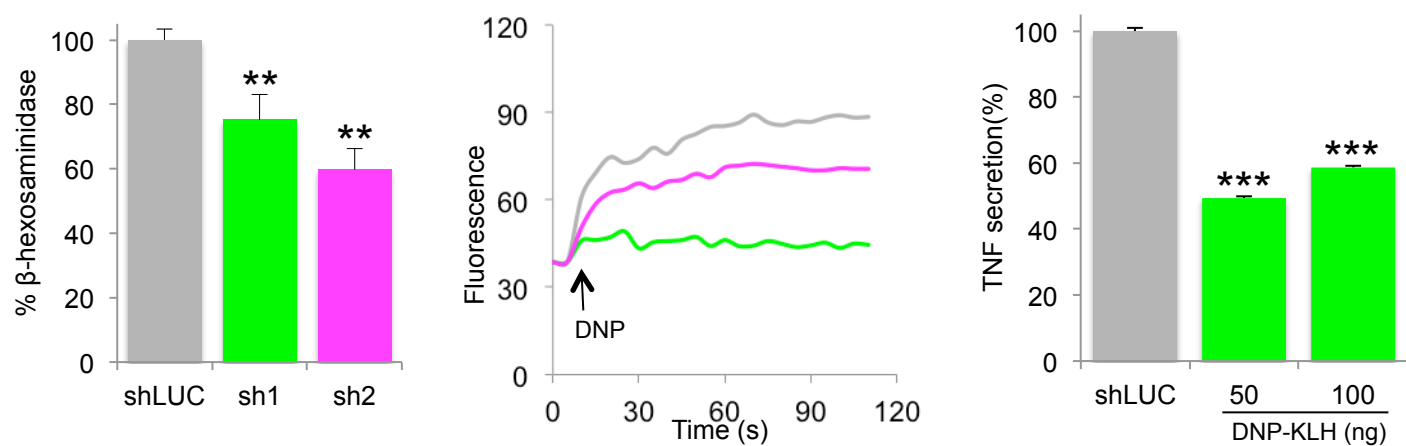

Supplement: Figure S8 — shRNA-induced down-regulation of C12orf4 expression. a) Two shRNA against rat C12orf4 (sh1 and sh2) were cloned in a retroviral vector and transduced in RBL-2H3 cells. Analysis of C12orf4 expression by qPCR and western blot were performed 10 and 15 days post-infection. b) Analysis of β-hexosaminisase release (left), calcium flux (middle), and TNFα secretion (right) were performed with cell populations 5 days post-infection. c) Western blot analysis of the FcεRI-mediated phosphorylation of major proteins implicated in mast cell activation. Cell populations transfected with sh1 C12orf4 (5 days post-infection) are compared with a control shRNA (shLUC), either non activated or activated with IgE/DNP for 3 and 10 minutes. **: p<0.01; ***: p<0.001 (t-test). (PDF) [file pone.0104998.s008.pdf]
