# Supplementary material for: In-Cell Intrabody Selection from a Diverse Human Library Identifies C12orf4 Protein as a New Player in Rodent Mast Cell Degranulation
Source: PLoS One. 2014 Aug 14;9(8):e104998. doi: 10.1371/journal.pone.0104998 (PMC4133367; doi:10.1371/journal.pone.0104998)
Supplement: Figure S2 — Individual clone phenotypes from plasmid library selection. a) 133 stable clones were tested for β-hexosaminidase release. Red dots represent the clones selected for sequencing. The clone 5H4, characterized in Fig. 3 of the manuscript, is marked. b) 36 clones were sequenced. Clones are sorted from the least to the most degranulating clone in the initial screen in (a). Nb seq: number of intrabody sequences retrieved by PCR. “> = 2”: the clone contains more than 1 sequence but was not analyzed further to determine the exact number of inserted intrabody. H3: VH CDR3 sequence. L3: VL CDR3 sequence. *: stop codon. x: unread because of poor sequencing quality. c) Clones for which a sequence was determined, were re-tested for β-hexosaminidase release (between 2 and 6 replicates). *: p<0.05; **: p<0.01; ***: p<0.001 (Student t-test). Clones 6E8, 8D12 and 5F5 were used as negative controls. (PDF) [file pone.0104998.s002.pdf]

a

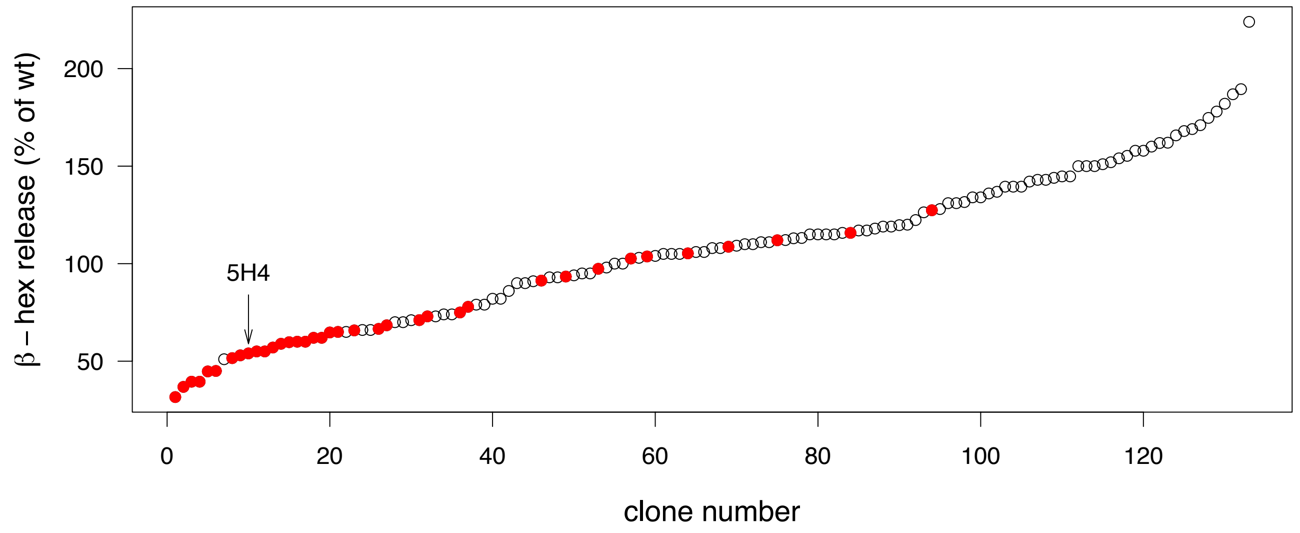

b

clone	β-hexo	Nb Seq	H3	L3
8F11	32	2	SSITIFGGGMDV	QQYCPSPST
			GDESLDW	QQYCPSPST
1D4	37	1	VMVFGEVDWSLDY	YTHGTTNTILF
3H2	39	1	PIAVSDY	QTYDGSRAV
7C3	39	1	frameshift in H3	
4D10	45	2	nr	QQVYNLPFS
			DCGYERIDH	*
3F7	45	1	VRPYNRMCECFEY	QPSFTTPFP
10C2	52	1	EGYVLDF	LSKHTTALV
8E4	53	>=2		
5H4	54	1	DGGLREGFDC	PS*
4E8	55	>=2		
5E2	57	>=2		
9D7	59	1	AREVVDFFFDQYDY	QQDYTHQRT
5F4	60	1	SSITIFGGGMDA	QQYYGTPFT
3B6	60	1	GRPRFDD	QQYFICSLP
6G10	60	>=2		
1E9	62	>=2		
4G5	62	1	DGVGFVAFGD	QQSYNLPYS
9G12	65	2	SSITIFGGGMDV	QQAYKKPFI
			CGTGVAEFDF	*
1B3	65	>=2		
1A10	66	2	GVGDVDT	QQYNSTPNT
			SSITIFGGGMDV	HQYNIMPQS
1G9	67	2	DVRSCLDY	PRYDSCSKLV
			EDSWTCNFDY	KTSDMRYTLSV
3B8	68	1	GRPRFDD	QQYFICSLP
4D8	71	2	NLFYDxxx	QHYRSAPLCI
			DGDVGHDY	EQYLSYLMI
2E2	73	1	NVSYGKIDY	QQYVNPFT
4A8	75	>=2		
2F6	78	1	frameshift in H3	
4B12	84	>=2		
6B11	91	1	AHKGFVIRSRFCYYYCLEV	ESCDSNSNSRV
3H10	93	1	GRPRFDD	QQYFICSLP
6D6	97	1	FAGGPEAFID	QTS
4H10	103	3	GGEDNNLRSRDH	QQSYSFPQT
			VVKACILDEVDY	*
			GGEDNNLRSRDH	*
10H5	104	1	frameshift in H3	
6E8	105	1	stop codon in H3	
10F8	109	>=2		
8D12	116	2	EDDCANVGDFFDN	QSWHGFNLV
			CDRAEKAHYCTDY	QLDNTKPRA
5F5	127	1	SSITIFGGGMDD	QQCYNYSLT

c

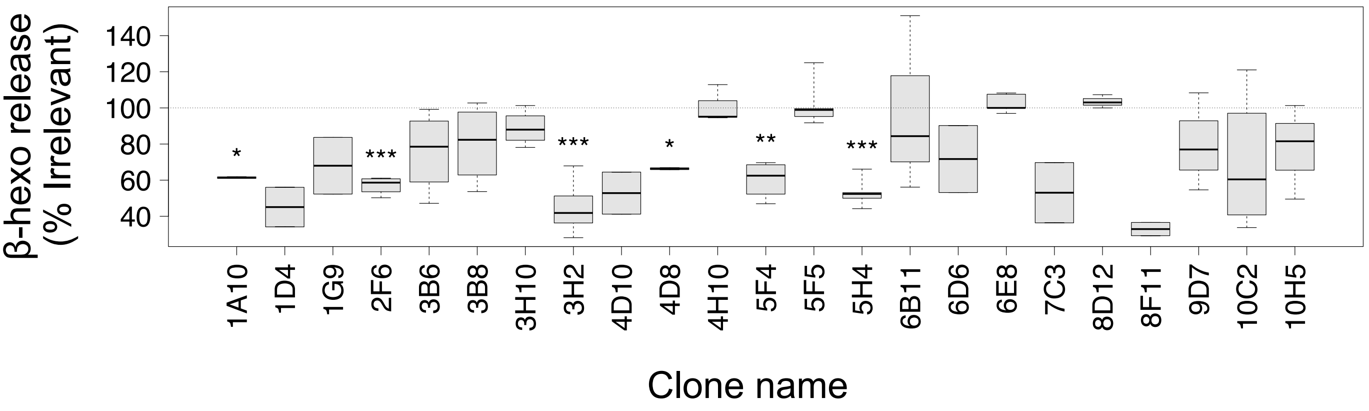
β-hexo release
(% Irrelevant)
140
120
100
80
60
40
*
***
***
*
**
***
1A10
1D4
1G9
2F6
3B6
3B8
3H10
3H2
4D10
4D8
4H10
5F4
5F5
5H4
6B11
6D6
6E8
7C3
8D12
8F11
9D7
10C2
10H5
Clone name
