# Supplementary material for: In-Cell Intrabody Selection from a Diverse Human Library Identifies C12orf4 Protein as a New Player in Rodent Mast Cell Degranulation
Source: PLoS One. 2014 Aug 14;9(8):e104998. doi: 10.1371/journal.pone.0104998 (PMC4133367; doi:10.1371/journal.pone.0104998)
Supplement: Figure S3 — Intrabody library diversity. Sequence analysis of DNA extracted from one million clones infected with the indicated retroviral libraries. Nb of reads: number of reads for each library; Nb of seq (dna): Number of different full length CDR3 DNA sequence; Nb of seq (dna no stop): Number of different full length CDR3 DNA sequence without stop codon or frameshift; Nb of seq (aa): Number of different CDR3 protein sequences obtained without stop codon or frameshift. a) VH CDR3. b) VL CDR3. Round3a and Round3 are the same pool but sequenced before and after recloning respectively (see Materials and Methods). (PDF) [file pone.0104998.s003.pdf]

a

VH

Libary	Nb of reads	Nb of seq (dna)	Nb of seq (dna no stop)	Nb of seq (aa)
Naive	10,962,195	456,029	363,616	244,467
Round 3a	1,090,966	32,079	26,655	20,194
Round 3	531,669	62,186	49,113	36,933
Round 5	834,599	38,500	29,266	21,592
Round 7	792,281	33,375	25,463	18,930
Round 8	787,691	35,793	27,092	20,223

b

VL

Library	Nb of reads	Nb of seq (dna)	Nb of seq (dna no stop)	Nb of seq (aa)
Naive	11,009,067	600,748	519,580	357,942
Round 3a	914,090	39,354	35,675	29,944
Round 3	563,580	74,572	67,506	54,876
Round 5	872,121	51,661	45,236	35,265
Round 7	824,388	52,565	45,893	35,743
Round 8	827,303	53,787	46,870	36,687
