# Supplementary material for: In-Cell Intrabody Selection from a Diverse Human Library Identifies C12orf4 Protein as a New Player in Rodent Mast Cell Degranulation
Source: PLoS One. 2014 Aug 14;9(8):e104998. doi: 10.1371/journal.pone.0104998 (PMC4133367; doi:10.1371/journal.pone.0104998)
Supplement: Figure S6 — Sequences of the most abundant retroviral clones from the 10 selected families. R_7 has a stop codon in the VL CDR3 loop and is thus truncated and expressed without the C-terminal eGFP tag. R_8 has the same VH than plasmid clone 5H4 and has been cloned as a single VH domain in retroviral vector. (PDF) [file pone.0104998.s006.pdf]

Clone	VH CDR3	VL CDR3
R_1	MDCVIGSYGYGIFDT	QSFVRNSTS
R_2	GKVLKKAEYSDWLDN	QQCSKFPLT
R_3	RSASCEH	EQYDTAPPYT
R_4	GEVGFDY	QQYFSQPFT
R_5	TLECSRCGDYGFDL	HQSNTYPFT
R_6	DGLYARMYYNGSYY	QQYFSQPFT
R_7	ERRDDDGMYAYSYQFDV	Q*
R_8	DGGLREGFDC	
R_9	NPASKCVYLEHDFEK	QTCNCLTLV
R_10	PERSAYDY	QQYSSHPLT
